# Supplementary figures and images for: Morphological and Genetic Assessment of Invasive Corbicula Lineages in Southern South America: A Case Study in Argentina
Source: Animals (Basel). 2024 Jun 21;14(13):1843. doi: 10.3390/ani14131843 (PMC11240789; doi:10.3390/ani14131843)

Estuarine clade

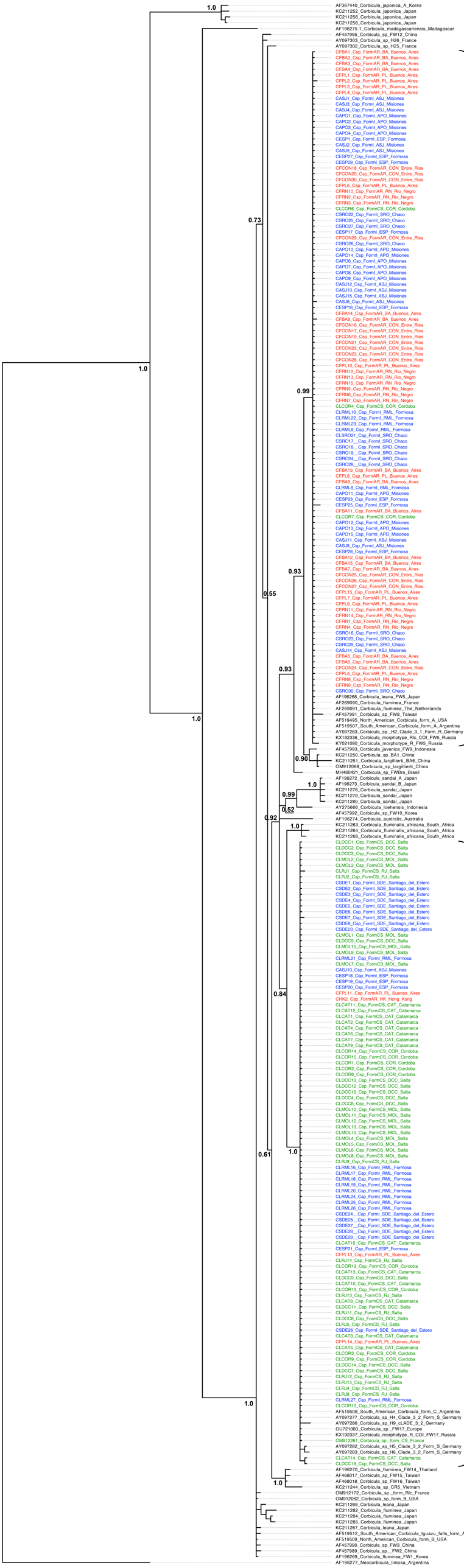

FW5

Freshwater clade

FW17

5%

Supplement: Supplementary file 1 [file animals-14-01843-s001.zip › Figure S1_mrbayes_tree_sin_colapsar_new.pdf]
